# High yield production of pigeon circovirus capsid protein in the *E. coli* by evaluating the key parameters needed for protein expression

**DOI:** 10.1186/1746-6148-10-115

**Published:** 2014-05-22

**Authors:** Guan-Hua Lai, Yen-Chang Lin, Yi-Lun Tsai, Yi-Yang Lien, Ming-Kuem Lin, Hsi-Jien Chen, Wen-Te Chang, Jason T C Tzen, Meng-Shiou Lee

**Affiliations:** 1 Graduate Institute of Biotechnology, National Chung Hsing University, Taichung, Taiwan; 2 Graduate Institute of Biotechnology, Chinese Culture University, Taipei, Taiwan; 3 Department of Veterinary Medicine, National Pingtung University of Science and Technology, Pingtung, Taiwan; 4 Department of Chinese Pharmaceutical Science and Chinese Medicine Resources, China Medical University, Taichung, Taiwan; 5 Department of Safety, Health and Environmental Engineering, Mingchi University of Technology, Taipei, Taiwan

## Abstract

**Background:**

Pigeon circovirus (PiCV) is considered to be a viral agent central to the development of young pigeon disease syndrome (YPDS). The Cap protein, a structural protein encoded by the *cap* (or C1) gene of PiCV, has been shown to be responsible for not only capsid assembly, but also has been used as antigen for detecting antibody when the host is infected with PiCV. The antigenic characteristics of the Cap protein potentially may allow the development of a detection kit that could be applied to control PiCV infection. However, poor expression and poor protein solubility have hampered the production of recombinant Cap protein in the bacteria. This study was undertaken to develop the optimal expression of recombinant full-length Cap protein of PiCV using an *E. coli* expression system.

**Results:**

The PiCV *cap* gene was cloned and fused with different fusion partners including a His-tag, a GST-tag (glutathioine-S-transferase tag) and a Trx-His-tag (thioredoxin-His tag). The resulting constructs were then expressed after transformation into a number of different *E. coli* strains; these then had their protein expression evaluated. The expression of the recombinant Cap protein in *E. coli* was significantly increased when Cap protein was fused with either a GST-tag or a Trx-His tag rather than a His-tag. After various rare amino acid codons presented in the Cap protein were optimized to give the sequence rCap_opt_, the expression level of the GST**-**rCap_opt_ in *E. coli* BL21(DE3) was further increased to a significant degree. The highest protein expression level of GST**-**rCap_opt_ obtained was 394.27 ± 26.1 mg/L per liter using the *E. coli* strain BL21(DE3)-pLysS. Moreover, approximately 74.5% of the expressed GST**-**rCap_opt_ was in soluble form, which is higher than the soluble Trx-His-rCap_opt_ expressed using the BL21(DE3)-pLysS strain. After purification using a GST affinity column combined with ion-exchange chromatography, the purified recombinant GST**-**rCap_opt_ protein was found to have good antigenic activity when tested against PiCV-infected pigeon sera.

**Conclusions:**

These findings shows that the *E. coli*-expressed full-length PiCV Cap protein has great potential in terms of large-scaled production and this should allow in the future the development of a serodiagnostic kit that is able to clinically detect PiCV infection in pigeons.

## Background

Pigeon circovirus (PiCV), is a non-enveloped virus and is considered to be the viral agent central to the development of young pigeon disease syndrome (YPDS). YPDS syndrome is a multifactorial disease that includes various unspecific clinical signs such as poor racing performance, weight loss, lethargy, anorexia, respiratory distress and diarrhea [1]. At present, PiCV is classified as a tentative member of circovirus family based on its particle size, its associated histopathology and the fact that it shares low-level DNA homology with psittacine beak and feather disease virus (BFDV) [2]. According to the previous reports on the genomic characterization of PiCV, PiCV has been characterized as having an ambisense single-stranded DNA genome of about 2.0 kb [3,4]. There are five open reading frames (ORFs) present on the ss-DNA; V1, C2, C3 and C4; these partially overlap within the PiCV genome. ORF C1 encodes a 30 kDa protein, which is the putative major component responsible for assembly of the viral capsid protein (Cap) [3]. ORF V1 encodes a non-structural protein with putative replication-associated protein (Rep) activity [4]. The ORFs C2, C3 and C4 encodes hypothetical proteins, the biological functions of which remain unclear. To date, some conventional methods have been used to detect the PiCV infection. These include electron microscopy, histological observation and molecular diagnosis including polymerase chain reaction (PCR), *in situ* hybridization and nucleic acid-based dot blot hybridization [5-10]. Enzyme-linked immunosorbent assay (ELISA) is a convenient and popular assay for diagnosis of virus infections and allows the investigator to target virus-specific antibodies in the sera of the host. Nevertheless, very few ELISA assay systems for detecting PiCV infection have been established successfully. Development of an ELISA system relies on the availability of viral antigens that are then used as ELISA coating antigen or for antibody production. However, the propagation of PiCV in cell culture has never been described, and harvesting viral antigen from pigeons is a tedious, ineffective and time-consuming process that results in a low yield. Thus, using a recombinant DNA method to express a PiCV viral antigen has been suggested to be a better strategy for the development of an ELISA assay system. In previously reports, only two expression systems have been used to produce PiCV Cap protein; these were a *E. coli* expression system and a baculovirus-insect cell expression system [11,12]. However, the production of the recombinant full-length Cap protein was found to be hampered in *E. coli* due to a failure to express the first 39 amino acid residues at the N-terminus of the Cap protein, the coding sequence of which includes a significant number of codons that are rarely used in *E. coli*. Moreover, in above system, most of the expressed recombinant Cap protein was found to be in the form of inclusion bodies [11]. The information available on the baculovirus-insect cell expression system for Cap protein expression in Sf9 does not provide information on productivity on a large scale that would allow further evaluation. In the above context, the *E. coli* expression system is still easier to carry out and is more cost-effective when applied to viral antigen production than the baculovirus-insect cell system, although the *E. coli* system does have some limitations.

To develop the Cap protein as coating antigen of an ELISA system, the above mentioned limitations associated with using an *E. coli* expression system need to be overcome; these include making sure that the full-length of the Cap protein is expressed in *E. coli* and using an expression system in which the majority of Cap protein is produced in a soluble form rather than as inclusion bodies. If successful, this would not only allow the efficient purification of capsid protein on a scale that would allow an investigation of PiCV structural biology but also the purified recombinant protein would be potentially useful when developing diagnostic kits for the clinical detection of PiCV infection.

In this study, the PiCV *cap* gene was fused to a series of different fusion tags in order to improve recombinant Cap (rCap) protein expression. The rCap was then expressed attached to three different expression tags in order to evaluate rCap fusion protein expression and production across a number of different *E. coli* strains. Three expression vectors were used, one harboring a glutathione-S-transferase (GST) tag, another harboring a 6xHis tag and finally, a third harboring a thioredoxin-6xHis (Trx-His); these were investigated to explore the effect of these very different fusion tags on the expression of rCap protein across various *E. coli* strains. In addition, optimizations of codon usage for various amino acids within the Cap gene were also carried out to give the rCap_opt_ sequence and then the effect of these changes on expression of rCap_opt_ in the various *E. coli* strains was assessed. Finally, purified rCap_opt_ protein was examined in order to determine its antigenicity and therefore its usefulness in further serodiagnostic applications. To the best of our knowledge, the yield of *E. coli* expressed full-length rCap_opt_ in this study after codon optimization of the *cap* gene is the highest known to date.

## Results

### The fusion tags and the strain preference falicitates the expression level of recombinant PiCV capsid protein in *E. coli*

**Figure 1:**
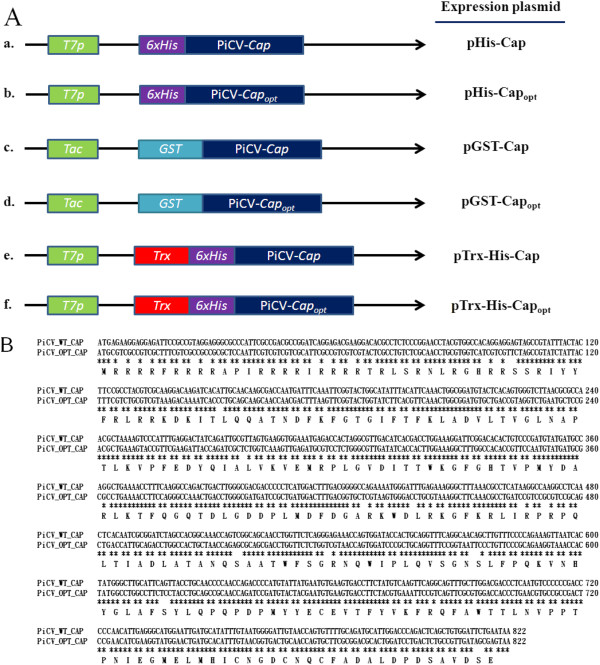
**A schematic diagram of the constructions used in this study and the alignment results for the expressed PiCV capsid gene. (A)** The full-length wild-type and codon-optimized PiCV capsid protein genes were cloned independently into three expression vectors pET28a, pGEX-4T-1 or pET32a. The PiCV capsid protein with the various different fusion tags, namely a six-histidine (6xHis), a Glutathione-S-transferase (GST) and a Thioredoxin-coupled six-histidine (Trx) at its N-terminus were expressed by *T7* or *Tac* promoter-driving after IPTG induction. **(B)** The nucleotide sequences were compared between the wild-type (WT) and the codon-optimized (OPT) PiCV capsid protein genes. The asterisk (*) represents the fact that the aligned nucleotides are identical.

The various fusion tags and the various *E. coli* strains had a range of effects on the expression level of the two recombinant PiCV capsid proteins in *E. coli* allowing optimization of the protein purification protocol. To investigate the expression of PiCV capsid protein (Cap) using the prokaryotic expression system, the *cap* gene sequence was individually fused with three different tag sequences, His-tag (6xHis), glutathione-s-transferase tag (GST) and thioredoxin-His (Trx-His) in three distinct expression vectors (Figure 1A, a, c and e). All above fusion tags were fused with recombinant rCap at its N-terminus. The resultant constructs, pHis-Cap, pGST-Cap and pTrx-His-Cap were then individually transformed into three distinct *E. coli* strains in order to address the effect of the fusion tags on the protein expression levels of the *cap* gene.

**Figure 2:**
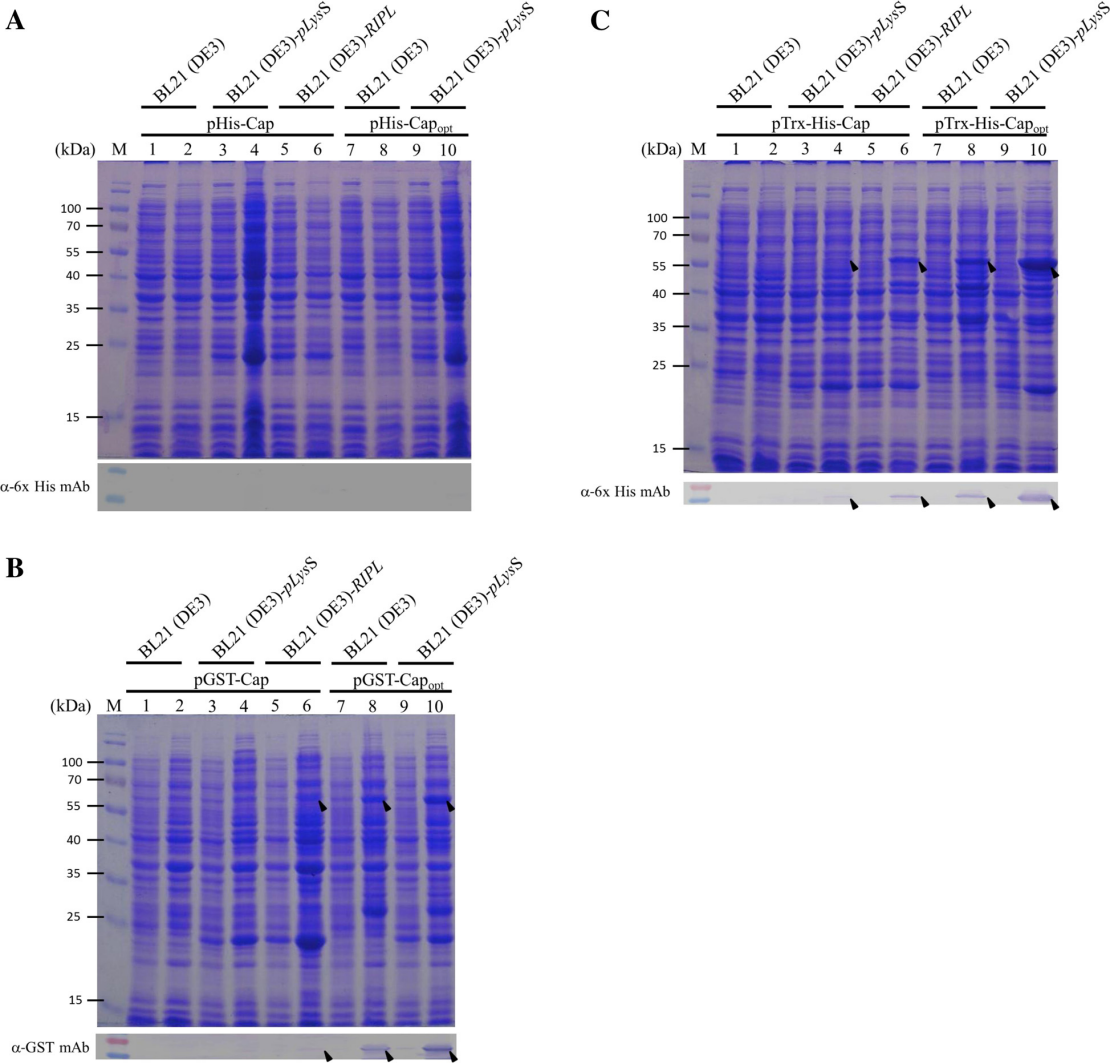
**Effect of fusion tags, *E. coli *strains and rare codon replacement on recombinant PiCV capsid protein expression.** The three expression plasmids containing wild-type PiCV capsid gene were expressed in *E. coli* strains BL21 (DE3), BL21 (DE3)-pLysS and BL21 (DE3)-CodonPlus-RIPL. Furthermore, similar plasmids but containing the codon-optimized PiCV capsid gene were also expressed in BL21 (DE3) and BL21 (DE3)-pLysS. All examined recombinant PiCV capsid proteins were analyzed by SDS-PAGE and Western-blotting. Anti-6xHis tag monoclonal antibody was used to recognize the 6xHis-tagged **(A)** and Trx-tagged **(C)** Cap proteins. Anti-GST monoclonal antibody was used for detecting GST-tagged **(B)** Cap proteins. Lane M, pre-stained protein marker; lane 1, 3, 5, 7 and 9, before IPTG induction; lane 2, 4, 6, 8 and 10, after IPTG induction for 4 hrs cultivation. The solid triangle pinpoints the expressed Cap protein.

As illustrated in Figure 2, when *E. coli* BL21(DE3) harboring pHis-Cap, pGST-Cap and pTrx-His-Cap were examined, there was no significant amount of rCap fusion protein present in the whole cell lysates after IPTG induction for 4 hrs (SDS-PAGE and Western-blotting of Figure 2A, lane 1–2; Figure 2B, lane 1–2; Figure 2C, lane 1–2, respectively). ). Proteins at the predicted molecular weights, 32 kDa His-rCap, 58 kDa GST-rCap and 48 kDa Trx-His-rCap, were not detected using anti-His monoclonal antibody and anti-GST antibody as appropriate (Figure 2A, lane 1–2; Figure 2B, lane 1–2; Figure 2C, lane 1–2 of Western blot).

As a result of the above findings, expression of the PiCV rCap fusion protein in *E. coli* was carried out in two other *E. coli* strains, BL21(DE3)-pLysS and BL21(DE3)-RIPL and their protein expression levels compared to that of *E. coli* BL21(DE3). The expression patterns of the rCap fusion protein in *E. coli* BL21(DE3)-pLysS and in *E. coli* BL21(DE3)-RIPL containing pHis-Cap, pGST-Cap, and pTrx-His-Cap are shown in Figures 2A, B, C, respectively,. The expression patterns for the rCap fusion protein were relatively poor in BL21(DE3)-pLysS. No matter whether the His-rCap or GST-rCap protein was being expressed, the protein products were almost undetectable. In contrast, the 48 kDa specific protein band for Trx-His-rCap could be detected using anti-His monoclonal antibody when the BL21(DE3)pLysS strain containing pTrx-His-Cap was induced with IPTG (Figure 2C, lane 3–4 of SDS-PAGE and Western blot). In addition, when pGST-Cap and pTrx-His-Cap were transformed into BL21(DE3)-RIPL strain, the GST-rCap and Trx-His-rCap protein could be successfully expressed and detected by both SDS-PAGE and Western-blot analysis (Figure 2B, lane 5–6; Figure 2C, lane 5–6 of SDS-PAGE and Western blot). Overall, expression level of the Trx-His-rCap protein, was significant higher than that of the GST-rCap protein in BL21(DE3)-RIPL strain. However, in the BL21(DE3)-RIPL strain containing the pHis-Cap plasmid, expression of His-rCap was still almost undetectable after IPTG induction (Figure 2A, lane 5–6 of SDS-PAGE and Western-blot, respectively). Thus the fusion tags GST and Trx-His seem to be able to facilitate expression of PiCV rCap protein in *E. coli* and, moreover, the *E. coli* strain used also plays a crucial role in expression and seems to affect the further application of these strains in large-scale production.

### Optimization of the codon usage of the cap gene enhances of recombinant PiCV capsid protein expression in E. coli

Based on the results shown in Figure 2A, B and C, the expression levels of rCap were improved in *E. coil* when a Trx-His tag or GST tag on the N-terminus of rCap protein was used. The best expression was obtained using the strain BL21(DE3)-RIPL, which harbors extra copies of tRNA^argU, proL, ileY, leuW^. This is commercial *E. coli* host strain used for the gene expression of recombinant proteins that contain *E. coli*’s rare codons. The expression of Trx-His-rCap protein in strain BL21(DE3)-RIPL showing an expression level was significant higher than when the BL21(DE3) and BL21(DE3)-pLysS strains were used (Figure 2B, lane 5–6; Figure 2C, lane 5–6), this suggested that optimization of the codon usage in the rCap protein might further improve the expression level of rCap.

**Figure 3:**
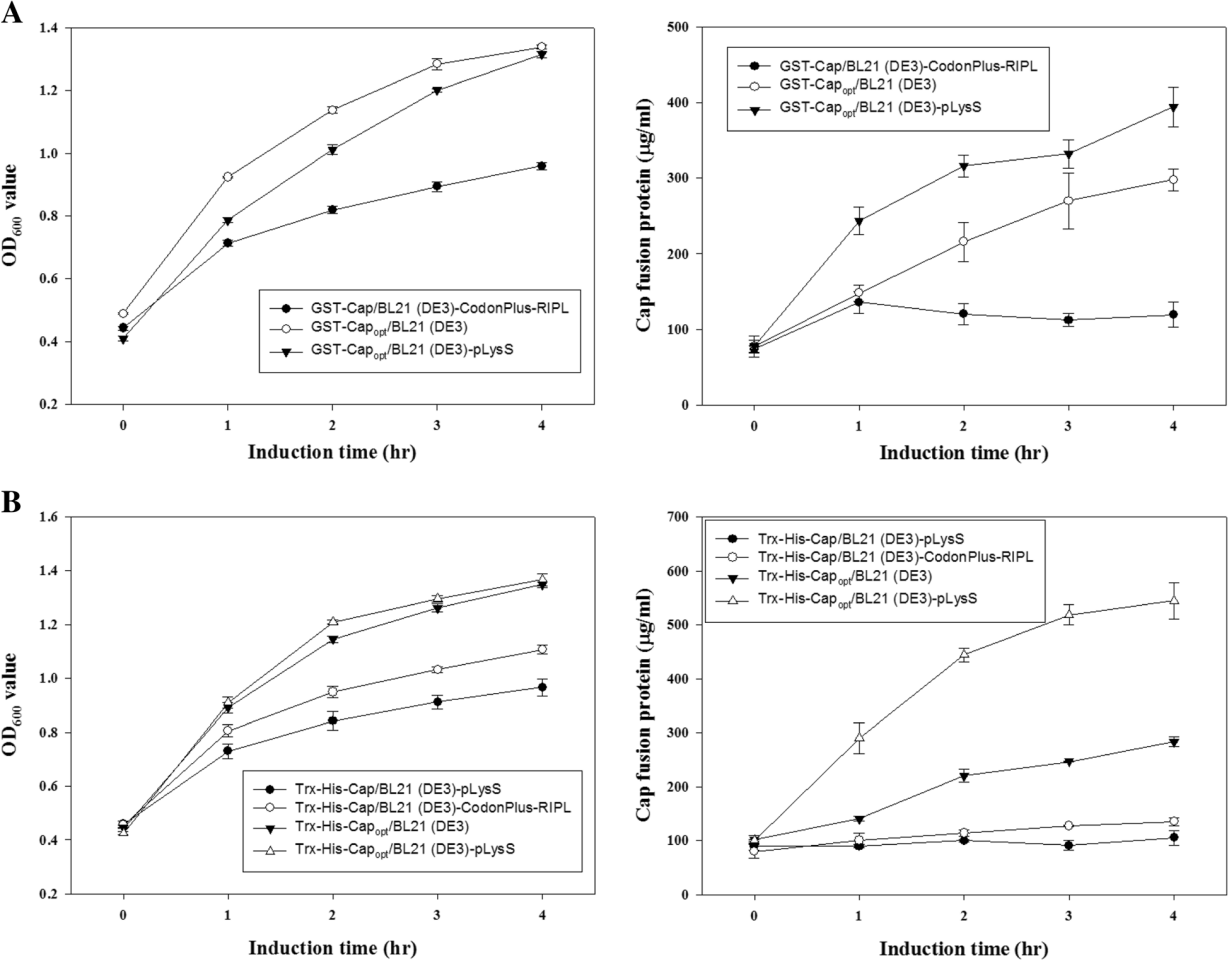
**Growth kinetics and production profiles of recombinant PiCV capsid protein (Cap) in different *E. coli *strains. (A)** The growth and protein production profiles of the three recombinant *E. coli* strains expressing either the GST-Cap or GST-Cap_opt_ protein. Two strains, BL21 (DE3) and BL21 (DE3)-pLysS expressing GST-Cap_opt_, and one GST-Cap expressing strain BL21 (DE3)-CodonPlus-RIPL were used in this study post-induction. **(B)** Growth and protein production profiles of four recombinant *E. coli* strains expressing Trx-His-Cap or Trx-His-Cap_opt_ protein. Two *E. coli* strains, BL21 (DE3) and BL21 (DE3)-pLysS were used to express Trx-His-Cap_opt_, and other two recombinant *E. coli* strains, BL21 (DE3)-CodonPlus-RIPL and BL21 (DE3)-pLysS were used to express Trx-His-Cap. Similar tests to those described above were used to detect the time course of protein expression after IPTG induction.

As illustrated in Figure 1B, the *cap* gene does contain a number of *E. coli*’s rare codons, which were detected when the sequence was examined by the GeneScript rare codon analysis tool (http://www.genscript.com/cgi-bin/tools/rare_codon_analysis). Approximately 18% rare *E. coli* codons are presented in PiCV *cap* gene, and most of rare codons are basic amino acid residues, such as lysine (K) and arginine (R) and are presented near the 5’-end of the *cap* gene. Using GeneOptimizer software, the codons of the *cap* gene were optimized without altering the amino acid sequence of the protein to give a gene sequence that had the preferred codon usage of *E. coli*. As illustrated in Figure 1B, this 819 bp DNA fragment was named as Cap_opt_. Three recombinant constructs were then created using the codon optimized *cap* gene sequence and then transformed into *E. coli* to explore expression levels (Figure 1A, panel b, d and f). As shown as Figure 2A, 2B and C, using SDS-PAGE and Western-blot analysis, when *E. coli* BL21(DE3) or *E. coli* BL21(DE3)-pLysS were used as host to express the His-rCap_opt_ or the GST-rCap protein, the expression proteins were almost undetectable after IPTG induction (Figure 2A, panel 7–8 for BL21(DE3) and panel 9–10 for BL21(DE3)-pLysS). However, when the recombinant *E. coli* BL21(DE3) and BL21(DE3)-pLysS strains harbored the pGST-rCap_opt_ plasmid, rCap_opt_ protein was produced at significant levels after IPTG induction (Figure 2B, panel 7–8 for BL21(DE3) and panel 9–10 for BL21(DE3)-pLysS to SDS-PAGE and Western-blot, respectively). The quantitative yield for GST-rCap_opt_ protein production using the BL21(DE3)-pLysS strain was 394.2 ± 26.1 µg/ml, which is higher than that of the GST-rCap protein at 119.2 ± 17.0 µg/ml when the latter protein is expressed in the BL21(DE3)-RIPL strain (right panel of Figure 3A and B), respectively. Moreover, the expression level of GST-rCap_opt_ protein in BL21(DE3)-pLysS was even higher than that of BL21(DE3) (Figure 2B, panel 7–10 to SDS-PAGE and Western-blot, respectively). This yield of GST-rCap_opt_ protein in the BL21(DE3)-pLysS was 1.3 fold higher than that of the GST-rCap_opt_ protein expressed in the BL21(DE3) strain (right panel of Figure 3A). Furthermore, the pTrx-His-rC_opt_ plasmid in the BL21(DE3) and BL21(DE3)-pLysS strains also produced a very similar pattern to that of the different *E. coli* strains expressing GST-rCap_opt_ protein (Figure 2C, panel 7–8 for BL21(DE3) and panel 9–10 for BL21(DE3)-pLysS to SDS-PAGE and Western-blot, respectively).

Furthermore, the quantitative yield for the Trx-His-rCap_opt_ protein in the BL21(DE3)-pLysS strain after IPTG induction for 4 h reached 544.5 ± 33.2 µg/ml at the same optical density (OD) as the cultures, which represents a 5.07 fold increase over Trx-His-rCap in BL21(DE3)-pLysS by densitometric analysis (Figure 2C, panel 3–4 for Trx-His-rCap and panel 9–10 for Trx-His-rCap_opt_; right panel of Figure 3B). In addition, the yield of Trx-His-rCap_opt_ protein in BL21(DE3)-pLysS was higher than that of Trx-His-rCap_opt_ protein at 283.3 ± 9.0 µg/ml using BL21(DE3) strain and IPTG induction (right panel of Figure 3C). These findings confirm that the codon-optimized of the *cap* gene was able to improve protein expression significantly and allowed large amounts of intact rCap_opt_ protein to be produced in *E. coli* BL21(DE3) or in BL21(DE3)pLysS with either the GST or the Trx-His fusion tag.

### Chromatographic purification of recombinant PiCV capsid protein

**Figure 4:**
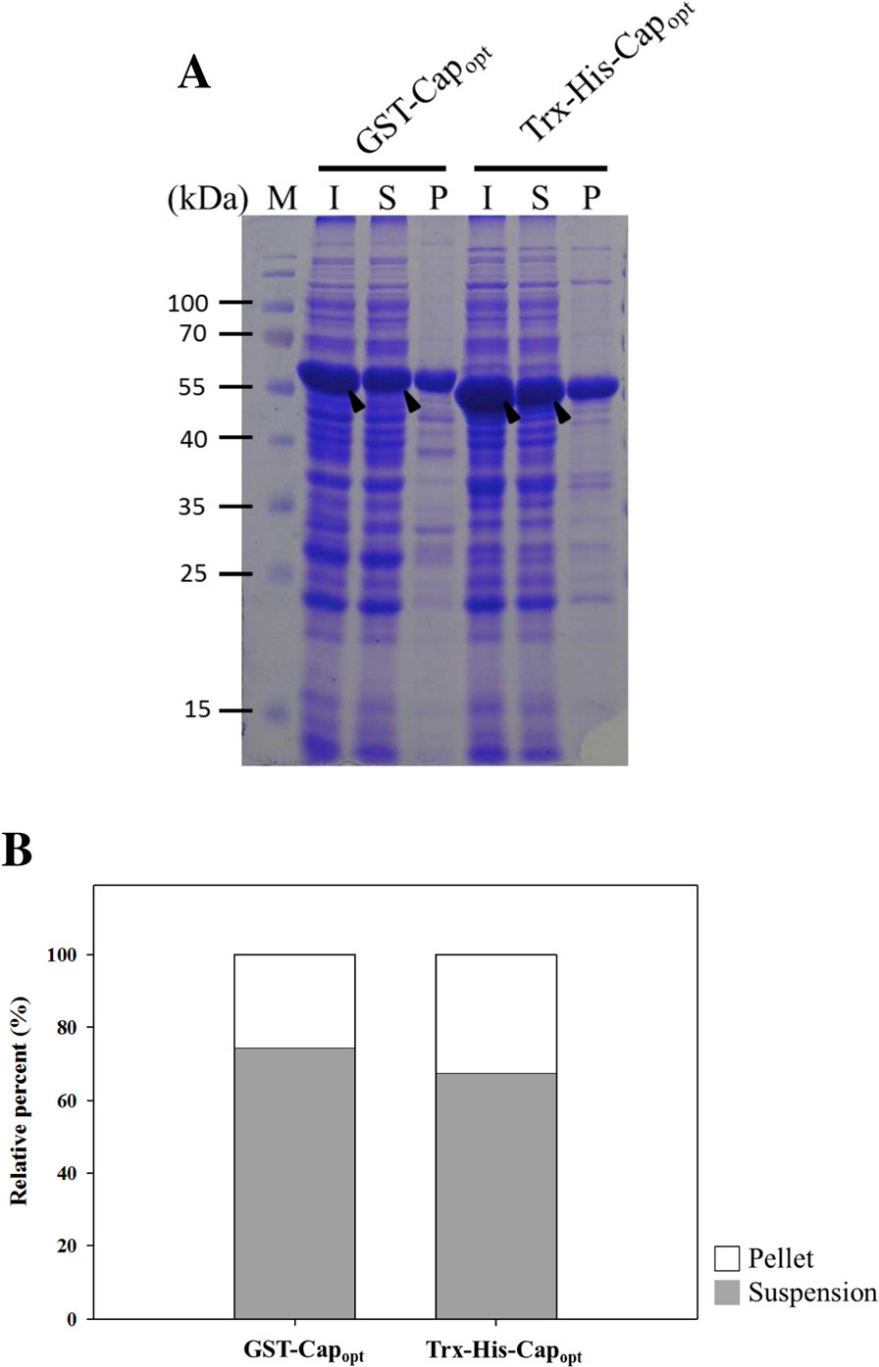
**The solubility of recombinant GST-Cap**_**opt **_**and Trx-His-Cap**_**opt **_**protein.** The two proteins were expressed by *E. coli* strain BL21 (DE3)-pLysS transformed pGST-Cap_opt_ and pTrx-His-Cap_opt_ respectively. SDS-PAGE **(A)** was used to analyze the protein distribution pattern of suspension fraction and the pellet fraction. The soluble percent of two recombinant proteins was determined by measuring the intensity of target protein bands on coomassie blue-strained gels **(B)**. Lane M, pre-stained protein marker; lane I, total protein-expressed cell lysate; lane S, suspension fraction from centrifugal protein-expressed cell lysate; lane P, pellet fraction from centrifugal protein-expressed cell lysate. The solid triangle pinpoints the expressed Cap_opt_ protein.

**Figure 5:**
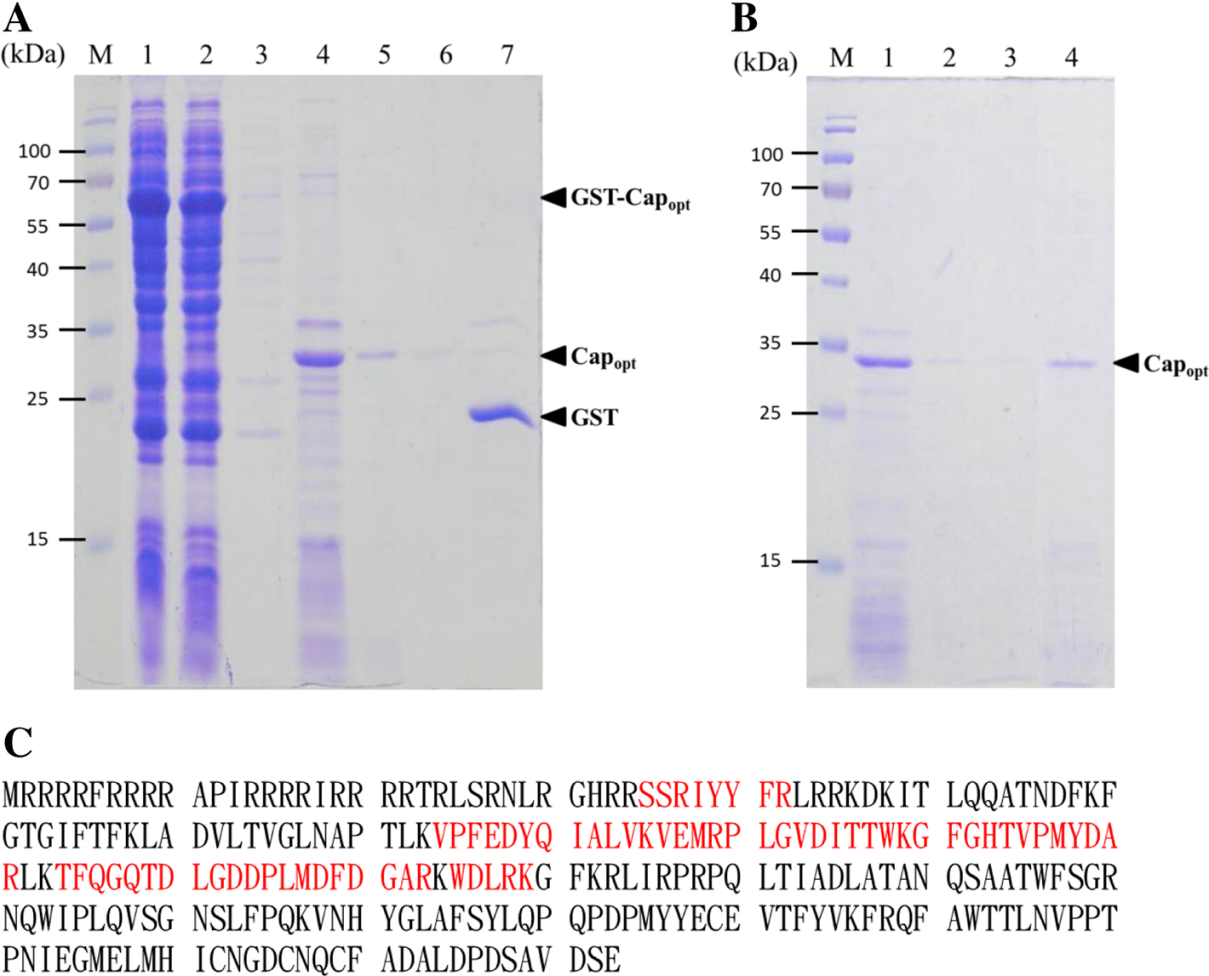
**Purification of recombinant PiCV-Cap**_**opt **_**protein using on-column cleavage by thrombin and SP cation exchange chromatography. (A)** The GST tag of recombinant GST-PiCV-Cap_opt_ protein was removed by the on-column cleavage method as described in Material and Method and the residue rCap_opt_ were eluted and collected for the next purified procedure. The results of the purification were examined by SDS-PAGE. Lane M, pre-stained protein marker; lane 1, total protein-expressed cell lysate; lane 2, flow-through, lane 3, GST affinity column washing; lane 4, Tag-free rCap_opt_ elute collected after on-column cleavage; lane 5, first column washing after on-column cleavage; lane 6, second column washing after on-column cleavage; lane 7 GST tag elute after on-column cleavage. **(B)** The rCap_opt_ eluted protein was further purified by SP cation exchange chromatography and the purification result assessed by SDS-PAGE. Lane M, pre-strained protein marker; lane 1, Input fraction of rCap_opt_ elute collected from a previous on-column cleavage purified step; lane 2 flow-through; lane 3, SP cation exchange column washing; lane 4, eluted fraction of purified rCap_opt_. **(C)** Identity of the rCap_opt_ protein was determined by MALDI-TOF. The red-labeled marker represents actual amino acid matches and there is 27% protein sequence coverage.

To further characterize and purify the rCap_opt_ protein, the solubility of expressed two rCap_opt_ fusion proteins, GST-rCap_opt_ and Trx-His-rCap_opt_ was explored. *E. coli* BL21(DE3)pLysS cells over-expressing either GST-rCap_opt_ or Trx-His-rCap_opt_ was separated into the supernatant and pellet fractions after sonication of a suspension of harvested cells. The analysis by SDS-PAGE demonstrated that both GST-rCap_opt_ and Trx-His-rCap_opt_ protein existed in soluble and insoluble forms (Figure 4A). By densitometric analysis, the solubility of GST-rCap_opt_ and Trx-His-rCap_opt_ protein were determined (Figure 4B). Approximately 74.58% of the GST-rCap_opt_ was soluble, which is higher than the 67.45% solubility obtained for Trx-His-rCap_opt_ protein when BL21(DE3)pLysS cells were used. Therefore, purification of the *E. coli*-expressed GST-rCap_opt_ protein was then carried using a GST affinity column. After affinity chromatography combined with on-column cleavage by thrombin, the presence of collected soluble rCap protein was clearly detectable by SDS-PAGE analysis (Figure 5A, lane 4). The specific 30 kDa band obtained from the column was approximately 90% pure (Figure 5A, lane 4), which indicates that rCap protein had been successful cleaved from GST fusion tag. To further improve the purity of the rCap, the GST-column purified rCap was subjected to cation exchange chromatography. As shown in Figure 5B, the purity of rCap was significantly increased by this process. Once the purification process had been completed, only cleaved GST fusion protein was present at almost homogeneity (Figure 5A, lane 7). When the chromatographic purified rCap protein was examined by MALDI-TOF, five peptides from rCap protein were identified after trypsin digestion and these demonstrated good alignment and a high score when compared to the predicted protein (Figure 5C). The longest peptide fragment, PLGVDITTWKGFGHTVPMYDAR consisted of 22 amino acid residues and, overall, the coverage was 27% of the published amino acid sequence of the PiCV Cap protein (Accession No. AER38484) without any miss-match (Figure 5C). These MALDI-TOF results confirmed that the purified 30 kDa protein is PiCV Cap protein and that the optimization of *E. coli*’s preferred codon usage within the *cap* gene had not altered the amino acid sequence (Figure 5C).

### Application of recombinant PiCV capsid protein on sero-diagnosis

**Figure 6:**
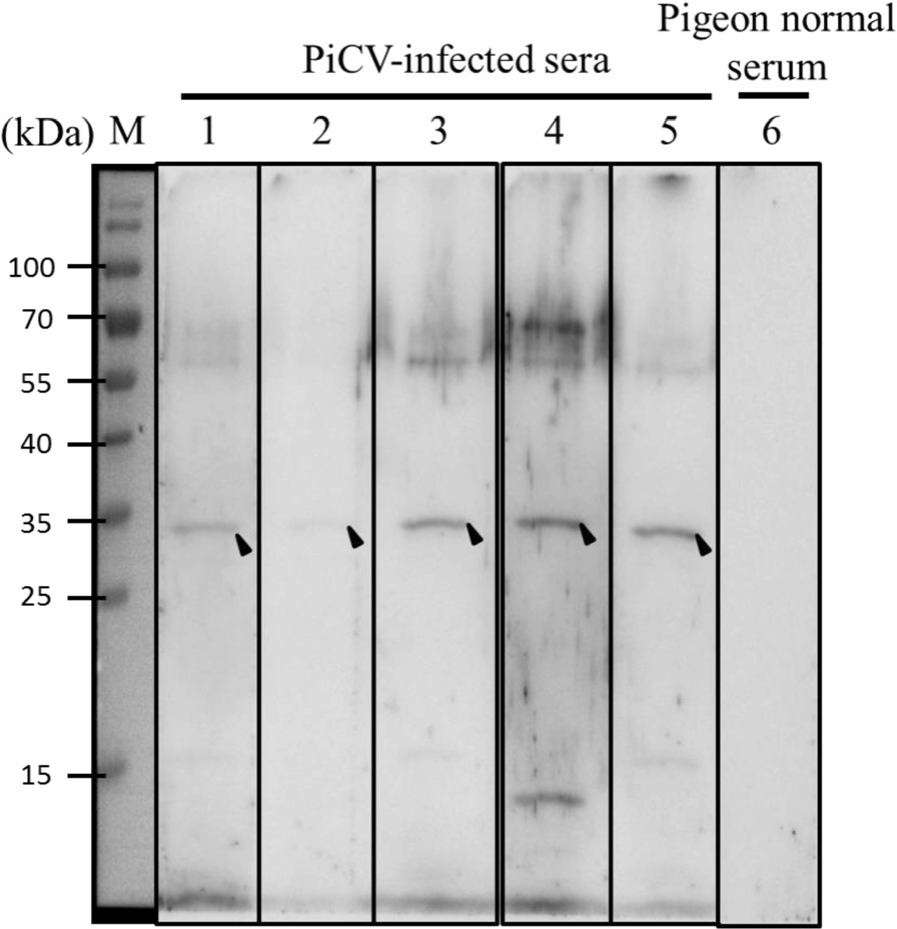
**Antigenicity characterization of the purified recombinant Cap**_**opt **_**protein by Western blot analysis using PiCV-positive sera.** Lane M, pre-stained protein marker; lane 1 to lane 5, PiCV-positive sera were used in Western blotting and the recognized position of rCap_opt_ protein is indicated by a solid triangle. The pigeons that had PiCV-positive sera were identified as virus-positive animals using a PCR detection methods as described in a previous study [6]. Lane 6, Pigeon normal serum was used as control.

Next we investigated whether the PiCV rCap protein expressed by *E. coli* has antigenic activity when against to PiCV-infected pigeon serum. As illustrated in Figure 6, Western blot analysis using PiCV-infected pigeon serum from five PiCV-infected pigeon showed that the *E. coli* expressed PiCV rCap proteins had the correct antigenic characteristics in terms of the detection of a specific band of approximately 30 kDa (Figure 6, lane 1–5). In contrast, when PiCV-noninfected serum was used, there were no any corresponding bands present on the PVDF membrane (Figure 6, lane 6). These findings supports *E. coli* expressed PiCV rCap_opt_ protein as retaining the protein’s original antigenic activity when used against PiCV-infected pigeon serum.

## Discussion

PiCV infection is associated with development of young pigeon disease syndrome (YPDS). At present, no vaccine is available to prevent PiCV infection. Among circovirus, capsid proteins have been investigated as to their usefulness as immunogens for developing subunit vaccines [13-15]. The Cap protein is the only capsid protein encoded by PiCV. Generally speaking, PiCV Cap protein is thought to be a promising target for the production of a recombinant vaccine or the development of a sero-diagnostic kit [11,12]. Recently, a truncated form of PiCV Cap protein has been shown to have been successful expressed in *E. coli*[11]. However, expression of full-length recombinant PiCV Cap protein using an *E. coli* system has remained very difficult.

*E. coli* remains the most attractive expression system when assessing the expression of a heterologous protein for many different purposes [16]. Using an *E. coli* expression system to express a heterologous recombinant protein has several advantages; these include cost-effectiveness, ease of production, time-saving and others. In this study, we have successfully produced for the first time the full-length Cap protein of PiCV using an *E. coli* expression system Previously, it has been suggested that the Cap protein is likely to be the sole structural protein of PiCV and this protein thus controls viral capsid assembly. Thus, have purified full-length Cap protein will help with the detailed study of PiCV's structure biology and it will also help with PiCV vaccine development and the production of a serodiagnostic kit for PiCV detection. The PiCV Cap protein has been demonstrated to have antigenic activity and to be able to recognize by PiCV specific antibodies [11,12]. However, up to the present, a lack of highly purified full-length Cap protein has hindered research in these areas. The main problem with the *E. coli* approach to producing PiCV Cap protein has been poor protein expression and low protein solubility. Previous studies have shown that the production of recombinant Cap protein using an *E. coli* expression is relatively difficult and therefore this has become a bottle-neck [11]. This study surmounts this problem and will allow the efficient production of recombinant PiCV Cap protein for future investigations.

A number of different strategies are available when improving protein expression and enhancing the protein solubility in *E. coli*. These factors include cultivation parameters, the fusing of an affinity tag of one type or another to the target protein and the optimization for *E. coli* of the codon usage of the foreign gene. [13,17,18]. This study explored how three different fusion tags, GST, His-tag and Trx-His tag, affected Cap protein expression; other fusion tag remain untested and may give further improvement in the future. Both a GST and a Trx-His fusion tag was found to significantly improve the yield of rCap protein compared to a 6 × His tag (Figure 2A, B and C). In a previous study, Liu *et al.* described how the expression of the Cap protein of porcine circovirus (PCV) was successfully improved in *E. coli* by fusing the maltose-binding protein (MBP) to the target protein [19], but the mechanism by which a fused MBP-8xHis tag is able to improve protein expression remains unclear. Nonetheless, one possibility is that protein solubility was improved [19]. Similarly, in our previous study, the addition of a GST tag to the CAV VP1 protein also improved expression in *E. coli* significantly compared to a His × 6 tag [13]. Thus some fusion tags would seem to be able to help improve the solubility of *E. coli* expressed proteins more than other tags; this occurs perhaps by promoting the correct folding of their selected fusion partner [13,19].

Like porcine circovirus (PCV), beak and feather disease virus (BFDV) and chicken anemia virus (CAV), PiCV is also rich in basic amino acid residues close to the N-terminus of the capsid protein. This region has been predicted to be a nuclear localizing sequence and a nucleic acid binding domain via the DNAbinder software package (http://www.imtech.res.in/raghava/dnabinder/submit.html). In previous studies, this N-terminal regions of capsid proteins have often caused problems with recombinant protein expression using a prokaryotic system [11,13,20]. Deletion of the N-terminus of the capsid protein can often overcome this problem allowing the protein to be expressed successfully in *E. coli*; nonetheless, the usefulness of the truncated protein for diagnosis or for the development of a subunit vaccine is likely to be hampered. Obviously only the intact capsid protein contains all of the epitopes for elicitation of virus neutralizing antibodies by the host. Thus, it is best to express full-length PiCV capsid protein rather than a truncated form when developing a vaccine or a diagnostic kit.

The present study found that the *E. coli* strain BL21(DE3)-RIPL, when used to express Trx-His-rCap, gave the highest level of protein expression, significantly higher than any other combination (Figure 2B, lane 5–6; Figure 2C, lane 5–6). This demonstrated that extra copies of tRNA^argU, proL, ileY, leuW^ are able to improve PiCV Cap protein expression usefully. In addition, it confirms that the *E. coli* rare codons with the *cap* gene have a significant effect on its expression. Using the GeneScript rare codon analysis tool, it was found that approximately 18% of the codons in the PiCV *cap* gene are rare *E. coli* codons. It had been suggested than when a target gene contains >10% rare cordons of *E. coli*, protein expression efficiency is likely to be decreased [21]. Rosenberg *et al.* also described how the efficiency of protein translation might be affected by an abundance of rare codons near the 5’-end of the gene. Thus, when a codon-optimized *cap* gene was used, rCap_opt_, rather than rCap, expression of the Cap protein was significantly enhanced in *E. coli* compared to supplying extra copies of the rare tRNA genes via the expression strain.

We also investigated which of two different recombinant *E. coli* strains, BL21(DE3) and BL21(DE3)pLysS, was able to improve protein production and yield. With both GST-Cap_opt_ and Trx-His-Cap_opt_, expression was better with BL21(DE3) and produced more Cap protein than BL21(DE3)-RIPL (right panel of Figure 3A and 3B). It is worth noted that BL21(DE3)pLysS has a higher growth rate than BL21(DE3) or BL21(DE3)-RIPL when expressing Trx-His-Cap_opt_ or Trx-His-Cap. (left panel of Figure 3B). This discrepancy may involve either higher protein stability or Trx-His-Cap_opt_ having a less cytotoxic nature when present in BL21(DE3)pLysS. However, these effect were not present during the production of GST-Cap_opt_ protein using *E. coli* BL21(DE3)pLysS at high expression levels. The superiority of Trx-His-Cap_opt_ expression in BL21(DE3)pLysS may be due to the presence in the strain of the *pLysS* plasmid during protein induction. The cytotoxicity tolerance of BL21(DE3)pLysS might be associated with the expression of T7 lysozyme which attenuates transcription leakage by T7 RNA polymerase. However, this phenomenon was not significant when GST-Cap_opt_ was expressed grown using BL21(DE3)pLysS. GST-Cap_opt_ was decreased when there was induction by IPTG. One possibility is that the cytotoxicity of GST-Cap_opt_ may be higher that that of Trx-His-Cap_opt_ in BL21(DE3)pLysS. In other words, a “protein burden” may not yet have been encountered when BL21(DE3)pLysS was used to express Trx-His-Cap_opt_. Overall, we concluded that BL21(DE3)pLysS is the preferred choice for expressing Trx-His-Cap_opt._

The solubility of recombinant Cap protein is a potential problem and in this context the solubility of GST-Cap_opt_ protein is superior to that of Trx-His-Cap_opt_. Furthermore, it is very easy to apply the protein to a GST-affinity column in order to carry out a protein purification. Moreover, the Cap protein contains basic amino acid residues rich at its N-terminus. Such highly positive charge amino acids within the recombinant Cap protein allow easy polishing by cation exchange column as part of down-stream processing. Therefore, it seems likely that, a higher purity of Cap protein can be obtained when an affinity column is combined with an ion-exchange chromatography during vaccine development.

In this study, positive PiCV-infected pigeon sera were used to evaluate the antigenic activity of the *E. coli*-expressed recombinant PiCV Cap protein. All tested PiCV-infected pigeon sera were able to demonstrate that *E. coli*-expressed recombinant PiCV Cap protein has the correct antigenic activities. This might be a result of the *E. coli*-expressed recombinant Cap protein displaying all of the appropriate protein antigenic regions on the protein surface for recognition by the pigeons' antibodies. However, rCap protein when used against some pigeon sera did not show very strong antigenicity. This perhaps suggests that the titers of antibodies against PiCV Cap protein have various levels in pigeons and such variation might explain in lower performance of antigenicity in certain birds.

## Conclusions

In conclusion, using a prokaryotic system, the optimal expression of recombinant full-length PiCV Cap protein was established successfully during this study. Furthermore, by fusing the Cap protein to an affinity tag, by using the appropriate preferred *E. coli* and by optimizing the codon usage of the polypeptide, it was possible to increase the yield of Cap fusion protein significantly. In this context, a convenient and cost-effective strategy for increasing the expression of Cap protein, which was used herein, was the direct engineering of the codons of the Cap protein to fit the *E. coli* codon preferences. This approach paves the way for the large-scale efficient production of the PiCV Cap protein. In the future, this will also allow recombinant Cap protein to be used as a potential antigen for the development of a PiCV diagnostic test.

## Methods

### Bacterial strains and cell inoculation

Three commercial *E. coli* strains, BL21(DE3) (Invitrogen, Carlsbad, CA), BL21(DE3)CodonPlus-RIPL (Stratagene, La Jolla, CA) and BL21(DE3)pLysS (Stratagene, La Jolla, CA) were used and maintained at 37°C in the Luria-Bertani (LB) medium (1% tryptone, 0.5% yeast extract, 1% NaCl, pH 7.0). First, 0.5 mL of an overnight culture was inoculated into 50 mL LB medium to allow strain activation by growth at 37°C for around 3 hours, by which time the optical density of culture had reach 0.5 of OD_600_. These bacterial cells were then used for transformation.

### Construction of the recombinant plasmids

A 822 bp of cDNA fragment consisting of the *cap* gene that encodes the full-length PiCV capsid protein was synthesized by Genemark Biosci & Tech Co. (Taichung, Taiwan) based on the published sequence (Columbid circovirus, isolate 9030; Accession No. AJ298229). This cDNA was cloned into either pET28a, pET32a (Novagen, Madison, WI) or pGEX-4T-1 (GE Healthcare, Piscataway, NJ) individually using *Eco*R1 and *Xho*I (Takara, Japan) restriction sites. The resulting recombinant plasmids were designated pHis-Cap, pTrx-His-Cap and pGST-Cap, respectively (Figure 1, panel a, e and c). To improve the codon usage of the *cap* gene from PiCV, a second cDNA sequence was synthesized by Genemark Biosci & Tech Co that contained the codons that were optimized for *E. coli*; this was also ligated individually into the same three *E. coli* expression vectors using the same restriction sites; these constructs were designated pHis-Cap_opt_, pTrx-His-Cap_opt_ and pGST-Cap_opt_, respectively (Figure 1, panel b, f and d). The six constructs were then individually transformed into One Shot^®^ Top10 (Invitrogen, CA) chemically competent *E. coli* for maintenance of the recombinant plasmids. Transformants that containing a insert of the correct size were then confirmed as correct by restriction enzyme digestion and by DNA sequence analysis.

### Expression of recombinant Cap protein (rCap) and codon optimized Cap protein (rCap_opt_) in *E. coli*

To express the recombinant rCap or rCap_opt_ protein, all the constructed recombinant plasmids carrying either the *cap* gene or the codon-optimized *cap* gene, as described in Figure 1, were transformed into various *E. coli* strains to allow evaluation of protein expression. Three commercial *E. coli* host strains, BL21(DE3), BL21(DE3)CodonPlus-RIPL and BL21 (DE3)pLysS, each with a different recombinant construction, were used for protein induction and expression. The culture conditions, the composition of the LB medium and the protein induction condition of these recombinant strains have been described previously [13]. After IPTG induction, samples of the cells were harvested and analyzed for protein expression. The total protein was measured by the procedure described in a previous study [22]. Samples containing the expressed Cap or Cap_opt_ proteins were analyzed by 12.5% SDS-PAGE and Western-blotting using a monoclonal anti-His antibody (Invitrogen, Carlsbad, CA) or a monoclonal anti-GST antibody (GE healthcare, Piscataway, NJ).

### Purification of recombinant Cap_opt_ protein using GST affinity chromatography with on-column cleavage by thrombin

Recombinant rCap_opt_ protein was purified from cells expressing the GST-rCap_opt_ protein. This was carried out by spinning down 50 mL of culture supernatant and resuspended the pellet in GST resin binding buffer (140 mM NaCl, 2.7 mM KCl, 10 mM Na_2_HPO_4_, 1.8 mM KH_2_PO_4_, pH 7.3). The mixture was then sonicated on ice three times for 3 minutes using a 20% pulsed activity cycle (MISONIX Sonicator^®^ 300). Next, the lysate was centrifuged for 10 min at 10,000 rpm to remove the cell debris. The resulting cell supernatant was loaded onto a GSTrap FF affinity column (GE healthcare, Piscataway, NJ) for protein purification using the standard procedure described in a previous study [13]. The total protein concentration of each collected fraction from the column was determined using a Micro BCA kit (Pierce, Rockford, IL) with bovine serum albumin acting as the reference protein. The purity of the protein from each fraction was analyzed by 12.5% SDS-PAGE and then the resulting gels were Western blotted using monoclonal anti-GST antibody (GE Healthcare, Piscataway, NJ).

### Mass spectrometry

To confirm the identity of the recombinant Cap_opt_ protein, *E. coli* expressed GST-rCap_opt_ protein that had been purified by GSTrap FF column was used. The rCap_opt_ protein that had been eluted from the GSTrap FF column was loaded onto a SP cation exchange chromatography column (GE Healthcare) for further purification. The cation exchange column-purified rCap_opt_ protein was then analyzed by 12.5% SDS-PAGE. The relevant band was then cut out from the 12.5% SDS-PAGE gel after coomassie blue staining and digested with trypsin. The resulting peptides were subjected to the MALDI-TOF-MS mass spectrometry (ESI-QUAD-TOF) to allow amino acid sequence identification of the protein, as described in a previous study [22].

## Competing interests

The author declares that they have no competing interests.

## Authors’ contributions

MSL participated in this study design, performed the experiments and in the writing of the manuscript. GHL performed the experiments, study design and participated in the construction of the plasmids. YYL participated in the experiments on protein antigenicity and MKL, YCL, and YLT participated in the protein purification step and determining protein solubility. JTCT participated in the data analysis and the writing of the manuscript. HJC and WTC coordinated the study and participated in performing ELISA assay. All authors read and approved the final manuscript.
